# Supplementary material for: Environmental Status of Cryptococcus neoformans and Cryptococcus gattii in Colombia
Source: J Fungi (Basel). 2021 May 24;7(6):410. doi: 10.3390/jof7060410 (PMC8225054; doi:10.3390/jof7060410)
Supplement: Supplementary file 1 [file jof-07-00410-s001.zip › jof-1182732-supplementary.pdf]

Table S1. Studies that reported the environmental conditions which favor the development of the fungus

**Species**	**Environmental conditions**									
	Precipitation (mm)		***Temperature (high mean temperature)***		***Evaporation***		***High solar radiation***		***Humidity***	
	+	-	+	-	+	-	+	-	+	-
C. neoformans	[83]		[83]				**[23]**		**[18]**	**[23]**
C. neoformans var grubii	**[81]**, [27]		**[81]**, [27], [22]				[56]		[22]	
*C. neoformans var grubii (*Serotype A*)*	[20], [56]	**[81]**	[26], [56]	[20], [56]			**[81]**		[26]	
C. gattii	[59], [19]	[83]	[19]	[83], [59]						
C. neoformans var. *neoformans*	[28], [29]		[28], [29]							
*C. gattii (*serotype B)	**[81]**, [56], [60]		[26]	[56], [60]		**[81]**		**[81]**, [56], [60]	[26], [56]	
C. gattii (serotype C)	[25], [24]	**[81]**	[25], [24], [26]	**[81],** [21]			**[81]**		[21], [24], [26]	

(+): Positive influence, (-): Negative Influence

In bold showed the studies performing statistical analysis.
